# IFN-γ Response Is Associated to Time Exposure Among Asymptomatic Immune Responders That Visited American Tegumentary Leishmaniasis Endemic Areas in Peru

**DOI:** 10.3389/fcimb.2018.00289

**Published:** 2018-08-21

**Authors:** Ivan Best, Angela Privat-Maldonado, María Cruz, Mirko Zimic, Rachel Bras-Gonçalves, Jean-Loup Lemesre, Jorge Arévalo

**Affiliations:** ^1^Instituto de Medicina Tropical Alexander von Humboldt, Universidad Peruana Cayetano Heredia, Lima, Peru; ^2^Laboratorios de Investigación y Desarrollo, Faculty of Sciences and Philosophy, Universidad Peruana Cayetano Heredia, Lima, Peru; ^3^Institut de Recherche pour le Développement (IRD), UMR177-INTERTRYP, Montpellier, France

**Keywords:** American Tegumentary Leishmaniasis, asymptomatic infection, cellular immune response, T cell proliferation, Th1 response

## Abstract

Clinical manifestations of American Tegumentary Leishmaniasis (ATL) include cutaneous (CL) and mucous forms (ML); however, there are asymptomatic individuals who despite being infected do not present any clinical manifestations. This study characterized the cell-mediated immunity of travelers who lived in the Andean highlands of Cusco, free of leishmaniasis transmission, which eventually visited leishmaniasis endemic in the Amazonian basin and returned home without any clinical signs of the disease. Their immune response was compared with CL and ML patients who acquired the disease during their stage in the same region. Fifty-four human subjects from the highlands of Cusco (Peru), who have visited an endemic area, were enrolled: 28 of them did not show any symptoms, 12 showed CL and 14 showed ML. Ten healthy subjects from a non-endemic area (HS) were included as controls. T-cell proliferation was evaluated using peripheral blood mononuclear cells (PBMC) stimulated for 5 days with a total soluble leishmanial antigen (TSLA) of *L. (V.) braziliensis*. Th1/Th2/Th17 cytokines were also quantified in the supernatants by a flow cytometry multiplex assay. T-cell proliferation was expressed as stimulation index (SI) and the cut off was fixed at SI >2.47. Fifteen out of 28 subjects did not show any signs of disease (54%); subjects with an SI above the cut off. They were defined as asymptomatic immune responders (AIR). CL and ML patients presented a higher SI than HS and AIR. Among the latter group, the exposure time to *Leishmania* was clearly associated with the IFN-γ response. Increased levels of this cytokine were observed in individuals who remained <90 days in an endemic area of leishmaniasis. Our results evidenced two sub-populations among asymptomatic individuals, one AIR who did not develop clinical disease manifestations when they were exposed to *Leishmania* in endemic areas. Exposure time to *Leishmania* in the wild was associated with the IFN-γ response.

## Introduction

American Tegumentary Leishmaniasis (ATL) is a zoonotic disease caused by parasites of genus *Leishmania* when people get in contact with infected sandfly vectors in the wild (Grimaldi and Tesh, 1993). The two most prevalent clinical forms of ATL in Peru are the cutaneous leishmaniasis (CL), locally known as Uta, and the severe mucocutaneous form (ML) called Espundia. The main species that causes ATL is *Leishmania (V.) braziliensis*, and is almost the only one associated to those 8–10% of CL patients, who later on developed the ML disease after several years of original skin lesions (Lucas et al., 1998; Davies et al., 2000). There is, however, a minority of patients who developed ML without a previous CL episode (Lindoso et al., 2009).

Among the infected people with *Leishmania*, not all will develop the disease (Biagi, 1953; Gonzalez and Biagi, 1968; Pampiglione et al., 1974; Follador et al., 2002; Fagundes et al., 2007a; Riera et al., 2008; Singh et al., 2014; Andrade-Narvaez et al., 2016). The asymptomatic category in the *Leishmania* infection was, many decades ago, proposed in both ATL and visceral leishmaniasis (VL) (Gonzalez and Biagi, 1968; Pampiglione et al., 1974).

The term asymptomatic in *Leishmania* infection was first used in 1953 in a Mexican cutaneous leishmaniasis area. Twelve out of 36 subjects, who showed a positive reaction to the Montenegro skin test (MST), did not develop ulcerated lesions or scars on the skin; those individuals were considered as asymptomatic carriers (Biagi, 1953). Similarly, another study in northern Italy first identified by MST, subclinical infection in an outbreak of human visceral leishmaniasis (Pampiglione et al., 1974). These results indicated that asymptomatic individuals were able to either clear the *Leishmania* pathogen or to host the parasite in a cryptic stage (Follador et al., 2002). The latter possibility was supported by reports which demonstrated the presence of parasite DNA among people who lived in VL endemic areas, but who never developed any clinical signs (Martín-Sánchez et al., 2004; Alborzi et al., 2008). The proportion of asymptomatic subjects could represent a large proportion of individuals living in cutaneous leishmaniasis endemic areas. A Tunisian study showed that 75% of healthy individuals, without a localized cutaneous leishmaniasis (LCL) history caused by *L. major*, were positive for a leishmanin skin test (LST) (Sassi et al., 1999). A previous study carried out in Peru, showed that 17% (16/94) of all infections caused by *L.(V.) peruviana* and evaluated by MST, corresponded to subclinical infections (Davies et al., 1995).

Defining Leishmania asymptomatic infections is very difficult because of the lack of a reliable biomarker. It is also not clear how one can discriminate parasite persistence in an asymptomatically infected individual from new infections that occur after the first episode, i.e. former parasites cleared by the immune response followed by new infecting parasite populations that will follow the same fate. Concerning asymptomatic biomarkers, a positive MST has been used as an indicator of host cell-mediated immune response against the parasite (Nogueira et al., 2008) and for the detection of asymptomatic or subclinical infection in endemic areas of leishmaniasis. However, it cannot make a distinction between active, inactive or past infections (Vega-López, 2003). There is evidence that biomarkers such cytokines and chemokines favor the identification of asymptomatic subjects in endemic areas of leishmaniasis (Sassi et al., 1999; Bittar et al., 2007; Ibarra-Meneses et al., 2017).

Concerning the limitations of having true asymptomatic individuals, the Cusco region in Peru offers a particular opportunity to study true asymptomatic cases. Members of Andean native communities living at 3,600 meters above sea level around Cusco, an area free of Leishmania transmission, descend to the Amazonian basin and become temporarily exposed to *Leishmania* parasites for a discrete and short period of time to carry out seasonal work or tourism activities, or they colonize leishmaniasis endemic areas. Many of the latter group returns to the highlands after several years living in contact with the *Leishmania* transmission cycle. Those individuals who return to the highlands, if they are *Leishmania* infected, are ideal to follow up and establish those cases that are true asymptomatic.

In this work we evaluated cellular immune response parameters, including inflammatory cytokines, in a group of individuals from the highlands of Cusco who went to the Amazonian basin, being exposed temporarily to leishmaniasis endemic areas. After a period of time the subjects returned to the highlands of Cusco where they were recruited to determine if there were asymptomatic immune responders (AIR) among them. Their T cell proliferation and cytokine production profile were compared with CL and ML patients. Furthermore, we assessed if these immune parameters were associated to one or more clinical or epidemiologic variables.

## Materials and methods

### Ethics statement

The study was approved by the Institutional Ethics Committee of the Universidad Peruana Cayetano Heredia (Registration number: 53892), and a written informed consent was obtained from all participants.

### Study population

**Figure 1:**
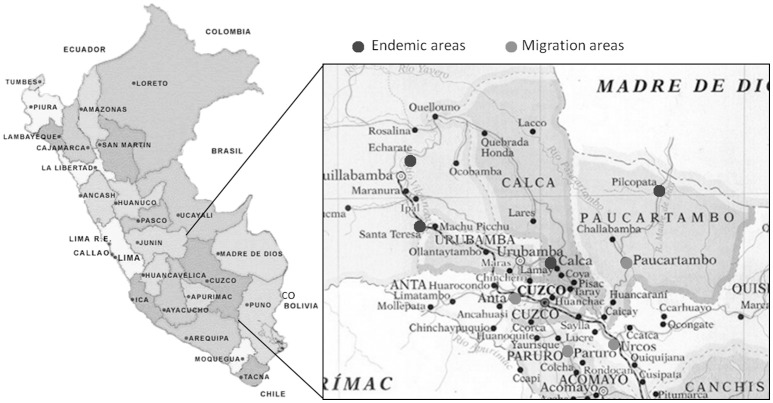
Endemic areas of American Tegumentary Leishmaniasis in Peru. Map showing the areas de migration from the highlands of Cusco to the endemic areas of American Tegumentary Leishmaniasis (ATL).

The study was conducted at the Instituto de Medicina Tropical Alexander von Humboldt of the Universidad Peruana Cayetano Heredia, Lima, Peru, where the blood samples obtained from individuals living in Cusco arrived within 6 h after their bleeding, being processed the same day for the immunological assays. The study groups consisted of 12 patients with active CL, 14 with active ML, 28 individuals that visited leishmaniasis endemic areas without any disease manifestation and 10 healthy subjects from Lima who never exposed to ATL endemic areas (HS). Individuals from Cusco without clinical manifestations were recruited among habitants of the highlands of Cusco (Canchis, Paruro, Urcos, Anta, and Paucartambo). They were aware that leishmaniasis is a disease that was transmitted at the areas they had visited and therefore were concerned about a possible infection with *Leishmania* parasites. These subjects might have been exposed to ATL endemic areas in the neighboring forests of Cusco and Madre de Dios (Pilcopata, Calca, Santa Teresa, Echarate) due to tourism, temporary residence or seasonal economic activities (Figure 1).

All CL and ML patients had a confirmed diagnosis of leishmaniasis by visualization of *Leishmania* amastigotes in Giemsa-stained smears, parasite culture and/or PCR test according to previous diagnostic procedures (Boggild et al., 2010). All the recruited individuals were negative to human immunodeficiency virus, hepatitis B surface antigen, hepatitis C virus, diabetes, anemia, pregnancy, and tuberculosis. None of them declared to have received corticoids treatment.

### Isolation of mononuclear cells

Peripheral blood mononuclear cells (PBMC) were isolated from Lithium Heparin-anticoagulated peripheral blood via density gradient centrifugation on Ficoll-Hypaque (GE Healthcare, UK), washed two times with Hanks's buffered salt solution (Gibco, USA), and one time with RPMI-1640 medium (Gibco, USA). All cells were resuspended in RPMI-1640 medium (Gibco, USA) and supplemented with 10% normal pooled human serum, 100 IU/ml penicillin (Gibco, USA), 100 mg/ml streptomycin (Gibco, USA), 2 mM L-glutamine (Gibco, USA), 1mM sodium pyruvate (Gibco, USA) and 1 mM non-essential amino acids (Gibco, USA); further referred to as complete medium. Cell viability was assessed by trypan blue dye exclusion.

### Preparation of *leishmania* antigens

Total soluble *Leishmania* antigen (TSLA) was prepared as follows: *L. (V.) braziliensis* (MHOM/BR/75/M2904) promastigotes (10^9^) were resuspended in 1 ml of lysis buffer [100 ul of 20x protease inhibitor cocktail (Sigma-Aldrich, USA), 1 mM PMSF, 2 mM EDTA pH 7.4, 1 mM Tris HCl pH 7.4]. Next, the *L. (V.) braziliensis* parasites were disrupted by ten repeated freezing and thawing cycles (1 min at −70°C and 2:30 min at 37°C), then sonicated at 60 Hz. The mixture was centrifuged at 14,000 rpm for 10 min at 4°C. The supernatant was stored at −70°C until use. A small sample was kept to determine the protein concentration using the Qubit Protein Assay Kit (Invitrogen, USA) and SDS-PAGE was done to confirm the integrity of the isolated proteins.

### Proliferation assays

PBMC were cultured in 96-well flat-bottomed plates (Falcon, Becton Dickinson, USA) in complete medium at 2 × 10^5^ cells per well. The cells stimulated with 10 ug/ml TSLA, 10 ug/ml phytohemagglutinin (PHA) or complete medium alone were incubated at 37°C in a humidified 5% CO_2_ atmosphere for 5 days. Afterwards, 1 uCi [^3^H]-thymidine (Sigma-Aldrich, USA) was added to each well for the last 5 h of incubation. The cells were harvested on filter paper (Filtermat A, Perkin Elmer, Finland), washed extensively and then liquid scintillation mixture (Sigma-Aldrich, USA) was added. Incorporated [^3^H]-thymidine was measured with a 1205 Betaplate Liquid Scintillation Counter (Wallac, Finland). T-cell proliferation was expressed as stimulation index (SI) which is c.p.m. of stimulated cultures divided by c.p.m. of unstimulated cultures. The cut off (mean + 3 *SD*) for a positive response was fixed from SI of HS.

### Cytokine measurement

Supernatants from cell cultures were collected on day 5 and analyzed with a flow cytometry multiplex assay (BD CBA Th1/Th2/Th17, Pharmingen, USA) to determine the levels of seven cytokines: IL-17A, IFN-γ, TNF-α, IL-10, IL-6, IL-2, and IL-4.

### Statistical analysis

The chi-square test was used to analyze categorical variables and the Kruskal-Wallis *H*-test for continuous variables without a normal distribution. The correlation between the level of each cytokine and the time of permanence in the endemic area was estimated with the non-parametrical Spearman's rank correlation test. AIR were classified in two groups after considering the time of permanence in the endemic area: individuals who stayed less or equal than 90 days vs. individuals who stayed longer than 90 days.

If possible, the levels of cytokines were transformed in order to have a normal distribution, confirmed with the Shapiro-Wilk *W*-test of normality (Boston and Sumner, 2003). The effect of the time of permanence in the endemic area on the normally-distributed transformed level of cytokine was tested after adjusting gender and age in a multiple linear regression.

## Results

### Immunological definition of asymptomatic immune responders (AIR)

**Figure 2:**
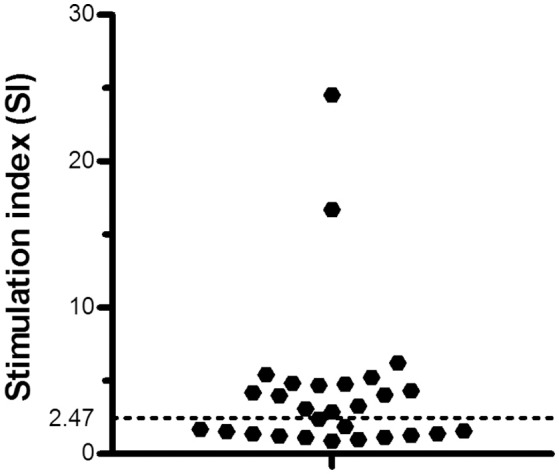
T cell proliferation in asymptomatic immune responders. Cut off for the T cell proliferation stimulation index (SI) in individuals exposed to an endemic area of American Tegumentary Leishmaniasis (ATL). Black dotted line limits the cut off.

Among the individuals who were exposed to ATL endemic areas but did not present any clinical manifestations, there were a set of individuals called AIR who were defined because of their SI were equal or above to 2.47. Fifteen out of 28 (54%) individuals who were temporarily exposed to the *Leishmania* transmission endemic areas showed significantly high SI (Figure 2). Therefore, SI was used to identify the AIR, the sub-population of individuals who were probably exposed to *Leishmania* antigens and therefore to *Leishmania* parasites infection but no disease clinical manifestation outcome occurred. For SI, the AIR showed a median of 4.7 while the corresponding non immune responders group a median of 1.4, respectively (*P* < 0.001, data not shown).

### Demographic characteristics of study population

According to the type of activity in the endemic areas, patients with active CL and ML were all male workers whereas the AIR group was composed by subjects who visited endemic areas for work or recreational activities, male and female individuals in equal proportions. The CL patients were significantly younger (*P* < 0.05) than AIR, ML patients and HS (*P* < 0.01, Table 1). No significant differences were found among these groups regarding the exposure time in the infection place (Table 1). Concerning the individuals that visited the leishmaniasis endemic areas there were not significant differences between AIR individuals and those ones who did not respond to *Leishmania* antigens regarding age, sex, occupation and time of exposure in the infection place (data not shown).

**Table 1:** Epidemiological parameters and immunological markers in cutaneous and mucosal leishmaniasis patients, asymptomatic immune responders and healthy subjects.

	**Healthy controls (*n* = 10)**	**Asymptomatic immune responders (*n* = 15)**	**CL patients (*n* = 12)**	**ML patients (*n* = 14)**	***P-value***
**EPIDEMIOLOGICAL PARAMETERS**					
Age in years^a^	28 (23.5–45.5)	36 (24.5–43)	19 (18–29)	40 (31.5–46.25)	0.005^*^ 0.2^**^
Male gender^b^	3 (30)	7 (47)	12 (100)	13 (93)	0.01^*^ 0.02^**^
Exposure time in an endemic area^a^		90 (37–1871)	180 (120–810)	378 (158–2051)	0.8^*^ 0.4^**^
Occupation^b^ Tourism Temporal residence/seasonal work		7 (47) 8 (53)	0 (0) 12 (100)	0 (0) 14 (100)	0.05^*^ 0.01^**^
**IMMUNOLOGICAL MARKERS**					
Stimulation index (SI)^a^	1.26 (0.79–1.63)	4.70 (3.98–5.40)	78.7 (30.8–178.3)	24.55 (14.65–71.47)	<0.001^*^ <0.001^**^
IFN-γ (pg/ml)^a^	3.35 (0–5.63)	16.10 (0–39.01)	3233 (1065–4057)	4451 (2111–8433)	<0.001^*^ <0.001^**^
TNF-α (pg/ml)^a^	1.3 (0–8.61)	6.60 (0.50–14.12)	102.6 (37.8–220.5)	142.4 (70.5–207.7)	<0.001^*^ <0.001^**^
IL-10 (pg/ml)^a^	3.7 (0–52.2)	27 (4.60–52.63)	53.9 (16.6–140.2)	77.9 (15.4–119.4)	0.083^*^ 0.037^**^
IL-17A (pg/ml)^a^	0 (0–7.8)	0 (0–7.20)	10.87 (4.78–14.98)	8.1 (0–32.1)	0.025^*^ 0.051^**^
Ratio IFN-γ/IL-10^a^	0.02 (0–0.69)	0.29 (0–0.74)	30.9 (21.8–74.3)	56.7 (31.5–133.6)	<0.001^*^ <0.001^**^

* *P-value for the comparison between CL patients and asymptomatic immune responders*,

** *P-value for the comparison between ML patients and asymptomatic immune responders*.

a *Age, exposure time in an endemic area, stimulation index, ratio IFN-γ/IL-10 as well as IFN-γ, TNF-α, IL-10 and IL-17A levels are presented as median (Q1–Q3)*.

b *Male gender and occupation is presented as absolute numbers and percentages (between brackets). CL, cutaneous leishmaniasis; ML, mucosal leishmaniasis; Q1–Q3, first quartile–third quartile*.

### Association of epidemiological parameters with the immune markers in the asymptomatic immune responders (AIR)

No statistical significance differences were observed between the individuals that visited the leishmaniasis endemic areas without clinical manifestations and HS for all tested cytokines. There was however a trend toward higher IFN-γ levels when compared AIR with HS (*P* = 0.071, Table 1). No significant differences were found when compared AIR with corresponding non-immune responders group (data not shown).

The level of IFN-γ in AIR showed a significant correlation with the time of permanence in an endemic area [Spearman's rho (ρ) −0.64; *P* = 0.010, *n* = 15, Table 2]. Individuals who stayed in the endemic area for a period longer than 90 days, showed significantly lower levels of IFN-γ (median = 1.75 pg/ml) than individuals who stayed there for a shorter period (median = 31.5, *P* < 0.05; Kruskal-Wallis *H*-test). In addition, the occupation of the inhabitants belonging to each of these groups was significantly different (*P* < 0.01, data not shown). Temporary residence or seasonal work like mining, agriculture, and construction were the principal occupations of the subjects who stayed in the endemic area for a period longer than 90 days while the main activity for individuals who stayed there for a shorter period was tourism (87.5%). The period of permanence in the endemic area was able to explain 35.8% of the variability of IFN-γ after adjusting the age and gender in the multiple linear regression (Table 3). No other cytokine showed a significant correlation. When cytokine levels were compared by gender among the different groups, no differences were observed in the levels of cytokines between females and males, except for a significant increase in TNF-α levels in females compared to males in the healthy control group (*P* < 0.05, data not shown).

**Table 2:** Spearman rank correlations between age, gender, IFN-γ, TNF-α, IL-10, IL-17A, ratio IFN-γ/IL-10, and exposure time in the infection place less or equal -and longer- than 90 days.

**Variables**	**Spearman's rho (ρ)**	*P*-value^*^
Age	0.3715	0.1728
Gender	−0.0714	0.8003
IFN-γ	−0.6866	**0.0047**
TNF-α	−0.3104	0.2602
IL-10	−0.2483	0.3722
IL-17A	−0.4536	0.0895
Ratio IFN-γ/IL-10	0.8189	**0.0002**

* *P-value for the comparison between subjects who stayed less or equal than 90 days vs. subjects who stayed longer than 90 days in an endemic area*.

**Table 3:** Univariate and multivariate comparison between subjects who stayed less or equal than 90 days and subjects who stayed longer than 90 days in an endemic area.

**Variables**	**Coefficient**	***P*-value**	**Coefficient**	***P*-value**
Age	−0.1139663	0.146	−0.0996245	0.164
Gender	−1.885137	0.235	−0.7773731	0.591
Exposure time in an endemic area	−0.0004683	**0.043**	−0.0004088	0.083

The square root transformation procedure converted the IFN-γ and the IL-10 into normally distributed variables (Boston and Sumner, 2003). The normalization was confirmed with the Shapiro-Wilk *W*-test (*P* = 0.55, 0.61; respectively). No other cytokine was able to be normalized by this statistic approach. The normally distributed and transformed IL-10 was not significantly associated, neither in the single nor in a multiple linear regression.

### T cell proliferation and cytokine response in CL and ML patients

As shown in Table 1, CL and ML patients presented significantly higher SI than AIR and HS (*P* < 0.001). Moreover, CL patients showed a significant increase of SI compared to ML patients (*P* < 0.01). After the evaluation of the effector response mediated by cytokines, a significant increase of IFN-γ and TNF-α production in patients with CL and ML at equivalent levels took place compared to AIR and HS (*P* < 0.001, Table 1). CL and ML patients presented higher IL-17A than AIR and HS. Patients with ML showed a significant increase of IL-10 comparing to AIR and HS (*P* < 0.05, Table 1). No differences in the remaining cytokines were observed among the different groups. In addition, CL and ML patients presented a significantly higher ratio IFN-γ/IL-10 compared to AIR and HS (*P* < 0.001).

## Discussion

The immune cell proliferation assay of PBMC obtained with *Leishmania* crude antigens discriminated between AIR and other individuals who went to the same disease endemic area but were unable to mount and/or keep an immune response. This discrimination was not feasible with Th1, Th2, or Th17 cytokines, although IFN-γ showed a trend to discriminate between AIR and HS. Therefore, MST still defines the asymptomatic status of an individual. It is highly sensitive but lacks specificity, as demonstrated by the occurrence of cross reactions with other diseases (de Lima Barros et al., 2005; Fagundes et al., 2007b).

Another contribution of this study is the population under study that offers an advantage to study truly asymptomatic individuals. In general, characterization of the cell-mediated immunity of asymptomatic patients may be obscured by the fact that they are usually exposed to recurrent *Leishmania* infection episodes because they are permanent residents in ATL endemic areas. This work exploited, however, a particular situation found on the highlands of Cusco, were members of Andean native communities became temporarily exposed to *Leishmania* parasites for a discrete period of time when they descended to the Amazon basin of Cusco or Madre de Dios. They stayed either for short periods of time to carry out seasonal work or tourism activities, or were temporal residents (up to 7 years, data not shown); however, each of them left the endemic transmission areas many years ago before being analyzed. It is within this population that is was possible to identify those individuals, who belonged to the AIR group, a true asymptomatic condition. Future studies on this population, with additional cellular immune response biomarkers should permit to define better profiles of asymptomatic individuals.

This study detected that 15 out of 28 subjects who traveled to the Amazon jungle of Cusco and/or Madre de Dios, showed a positive T-cell proliferation when challenged with *L. (V.) braziliensis* crude antigen. Here, they are called AIR and presented a significantly lower SI as well as a lower pro-inflammatory cytokines production (IFN-γ, TNF-α) compared to the strong T cell response, observed during active CL and ML (Table 1). The AIR responded on a past infection with a moderate but significant IFN-γ when challenged with *L. braziliensis* TSLA. Interestingly, an increased relative proportion of IL-10-producing cells, expressed by a low IFN-γ/IL-10 ratio, were observed in the AIR compared to active CL and ML which showed high IFN-γ/IL-10 ratios. The ratio between effector and regulatory specific T cells may influence the outcome of infection. Thus, these results lead us to propose that IL-10 in AIR could counter-regulate the IFN-γ effects, thus maintaining tissue integrity with an absence of ulcers or lesions. Our results were comparable with a previous report that found a low IFN-γ/IL-10 ratio in asymptomatic *Leishmania* carriers compared to cured CL patients (Bittar et al., 2007). The IL-10 protective role, here proposed in ATL, should be added to other immunological factors implied in host's resistant mechanisms (Díaz et al., 2010).

Previous studies, made in murine models, showed the role of the adaptive immune response mediated by CD4 Th1 and Th2 cells in the establishment and course of the *Leishmania* infection (Alexander and Bryson, 2005). IFN-γ produced by Th1 cells activates infected macrophages to eliminate the *Leishmania* by the production of nitric oxide (NO). However, an exacerbated production of this cytokine, observed during active CL and ML (Table 1), could be associated to tissue damage (Liew and O'Donnell, 1993; Roberts, 2006; Sharma and Singh, 2009; Silveira et al., 2009). On the other hand, IL-10 is now considered as a regulatory cytokine involved in the persistence of the parasite in the skin while it originally was included as a Th2 cytokine that inhibit the macrophage activation and proliferation of Th1 cells (Rodriguez et al., 2007).

Consistent with a study carried out in Brazil (Gomes-Silva et al., 2007), in our study, AIR had a significantly lower degree of antigen-specific T-cell expansion manifested by a lower SI and pro-inflammatory cytokines production (IFN-γ, TNF-α), in contrast to the strong response of T cells observed during active CL and ML (Table 1).

Nevertheless, when the effector T cell response between the AIR and HS was compared, the specific production of all tested cytokines were unable to discriminate these groups (Table 1). It was only possible to observe a significant association between IFN-γ response and the nature of *Leishmania* exposure when the IFN-γ production was measured in AIR individuals who spent less time in the infection place, mainly engaged to tourism activities. They showed increased IFN-γ levels respect to those individuals who have spent more time in the infection place due to seasonal works. These data suggested that the exposure time and activity type, which the migrants undertook in the Amazon jungle, could influence the nature of the immune response in the absence of primary transmission of ATL. The difference in the IFN-γ levels could be explained by the way these two sub-populations interact with the Amazon jungle. Individuals involved in tourism activities are probably more susceptible and more exposed to the transmission by leishmaniasis vectors; while seasonal workers develop immunity against the parasite through its probably less aggressive activity toward the forest and their greater exposure time in an endemic area. Consistent with this hypothesis, a study carried out in Bolivia showed that migrants from the highlands have an increased risk to develop CL and ML compared to the natives who lived in an endemic area (Alcais et al., 1997). Studies with other parasites reported that individuals, who lived in areas of lower malaria transmission, had higher IgG response to *Plasmodium falciparum* merozoite surface protein-1 (PfMSP1–19) compared to a neighboring village with higher malaria transmission (Braga et al., 2002).

We consider that AIR correspond to true asymptomatic carriers although it might be possible that some individuals would be either subclinical diseases or people who completely cleared the infection parasite (Biagi, 1953; Gonzalez and Biagi, 1968; Pampiglione et al., 1974; Follador et al., 2002; Martín-Sánchez et al., 2004; Fagundes et al., 2007a; Alborzi et al., 2008; Riera et al., 2008). To understand the biological basis why infected people do not develop disease requires a sustained and considerable number of volunteers, a situation found in the population living in the highlands of Cusco.

It is still unclear why some infected individuals, when exposed to an endemic area of ATL, develop disease while others do not present any clinical manifestations. The latter, here called AIR, could represent individuals with a natural resistance to develop ATL diseases. The development of an appropriate immune response, which controls parasite replication and maintains tissue integrity, is the simplest and straightest explanation for this phenomenon. There is however a new option to be incorporated in future studies, the quiescent stage of the *Leishmania* amastigote (Kloehn et al., 2015; Jara et al., 2017; Mandell and Beverley, 2017).

## Author contributions

IB, RB-G, J-LL, and JA conceived and designed the research. MC made the inclusion of patients. IB and AP-M performed the experiments. IB and JA analyzed the data and wrote the paper. All authors have read and approved the manuscript.

### Conflict of interest statement

The authors declare that the research was conducted in the absence of any commercial or financial relationships that could be construed as a potential conflict of interest.
